# Cervical cancer in pregnancy: Number 5 – May 2023

**DOI:** 10.1055/s-0043-1770142

**Published:** 2023-06-20

**Authors:** Geórgia Fontes Cintra, Sophie Françoise Mauricette Derchain, Delzio Salgado Bicalho, Agnaldo Lopes da Silva Filho, Walquíria Quida Salles Pereira Primo

**Affiliations:** 1 Instituto Brasileiro de Controle de Câncer, São Camilo Oncologia, São Paulo, SP, Brazil; 2 Universidade Estadual de Campinas, Campinas, SP, Brazil; 3 Instituto Oncoclínicas, Belo Horizonte, MG, Brazil; 4 Universidade Federal de Minas Gerais, Belo Horizonte, MG, Brazil; 5 Universidade de Brasília, Brasília, DF, Brazil

## Key points

- The incidence of cancer during pregnancy has increased given women's tendency to delay pregnancy. Cervical cancer is the third most commonly diagnosed neoplasm during pregnancy.
- Screening and diagnosis should be performed as in non-pregnant patients; cervical cytology is an obligatory antenatal exam, and colposcopy with biopsy may be performed at any time of pregnancy.
- Pregnancies complicated by the diagnosis of cancer should always be carried out in a reference center by a multidisciplinary team.
- The termination of pregnancy for standard treatment in specific situations is supported by law.
- Neoadjuvant chemotherapy is a safe alternative treatment during pregnancy to allow reaching fetal maturity. Response rates are high and neoplastic progression during pregnancy is reported in only 2.9% of cases. The risk of fetal malformations from chemotherapy is similar to that of the general population. However, chemotherapy is associated with intrauterine growth restriction, low birth weight, and neonatal myelotoxicity.
- In the absence of disease progression, the pregnancy should be carried to term.

## Recommendations

- Screening for cervical cancer in pregnant women should be the same as in other women.
- Treatment of precursor lesions (CINII or III) should be performed after delivery as the risk of progression during pregnancy is minimal.
- Staging of invasive lesions should preferably be performed using magnetic resonance imaging of the abdomen without contrast, and chest X-ray with abdominal protection.
- The therapeutic decision will take into account stage, gestational age and the patient's desire to maintain the pregnancy.
- There is legal support for terminating pregnancies in patients with cervical cancer and gestational age < 22 weeks to allow for standard care (surgery or radiotherapy with or without concomitant chemotherapy).
- The alternatives for patients who wish to preserve the pregnancy are: expectant management, conization/trachelectomy with or without pelvic lymphadenectomy or neoadjuvant chemotherapy with carboplatin and paclitaxel.
- In patients with invasive disease at the moment of delivery corporal (classical) cesarean incision is indicated. Vaginal birth is contraindicated in this scenario.
- After delivery, the patient should receive standard care according to the stage of the disease.

## Background

Cervical cancer is the most common gynecological cancer in pregnancy, with an estimate of 0.1-12 per 10,000 pregnancies. For cervical intraepithelial neoplasia, incidence rates range from 1.3 to 2.7 per 1,000. 1 2 3 Studies have not shown differences in the oncological prognosis of women with cervical cancer diagnosed during pregnancy compared to that in non-pregnant women. 1 2 3 The growing number of pregnant patients treated for a neoplasm and the follow-up of children resulting from these pregnancies generate safety in the use of various chemotherapy drugs during pregnancy. This has reflected in a greater number of pregnancies carried to term and better neonatal and neuropsychomotor development outcomes for these children. 1 3

### What is the conduct related to the pregnant patient with altered Pap smear?

Screening for pre-neoplastic lesions and cervical cancer in pregnant women should be performed using colpocytology following the recommendations for periodicity and age range for non-pregnant women. Visiting a health service for antenatal care should always be considered as an opportunity for screening. 1 4 Patients with altered cervical cytology should be referred for colposcopy. There is no contraindication for performing a biopsy at any stage of pregnancy. 4 In patients with a histological diagnosis of CINII or CINIII, treatment should be postponed until after delivery, given the minimal risk of neoplastic progression during pregnancy. Follow up with colposcopy every 12 weeks should be carried out. The biopsy should be repeated only if invasion is suspected. Diagnostic conization is indicated in pregnancy only if staging or confirmation of residual invasive disease change the timing and type of delivery. Otherwise, this procedure should be postponed to the postpartum period.

### What is the management of pregnant patients with a suspected lesion of invasive cervical neoplasia?

Suspicious cervical lesions in pregnant patients should be investigated through incisional biopsy. After confirmation of malignancy, imaging staging should preferably be performed using chest X-ray with abdominal protection and magnetic resonance imaging of the entire abdomen without contrast, considering that gadolinium is associated with rheumatological diseases in children and neonatal death. When magnetic resonance imaging is not available, an ultrasound of the entire abdomen can be performed with emphasis on the kidneys and urinary tract. 1 3 5

### Which centers are able to manage patients with cervical cancer diagnosed during pregnancy?

These patients should be treated in a reference center with a multidisciplinary team (oncological gynecologists, clinical oncologists, obstetricians specializing in high-risk pregnancies, neonatologists, radio-oncologists and psychologists). Treatment depends on staging, gestational age and the desire to preserve the pregnancy, always on an individual basis and after multidisciplinary discussion, taking into account the risks of postponing or modifying the treatment for that patient.

### Is it possible to legally terminate pregnancy in pregnant patients with cervical cancer?

It is impossible to carry out standard therapy for cervical cancer (radical surgery and/or pelvic radiotherapy) and maintain the pregnancy. Therefore, the termination of pregnancy up to 22 weeks in patients with cervical cancer is provided for in article 128 of the Brazilian Penal Code (necessary or therapeutic abortion when there is a risk to the mother's life) 6 and by Ordinance GM/MS number 1.508 from the Ministry of Health. 7 After this gestational age, the fetus is considered viable in most centers and the management must be individualized. Termination of pregnancy followed by standard oncologic treatment is recommended in patients with locally advanced disease or with positive lymph node. In this context, patients who choose to continue with the pregnancy should be informed they will not undergo standard oncological treatment, which could result in compromised maternal prognosis and increased obstetric risks. 1 3

### How should pregnancy be terminated?

For termination of pregnancy in patients with cervical cancer, the following are required:

- The evaluation of at least two professionals; one of them must be a specialist in the disease causing the interruption:
- Medical record with medical justifications detailing the maternal risk;
- The consent and/or informed consent signed by the pregnant woman or her family, unless this is impossible in situations of imminent risk to her life;
- Support and monitoring by a multidisciplinary team, especially psychologists.

Judicial authorization, police reports or communication to the Regional Council of Medicine are not required. The method of termination of pregnancy depends on gestational age and staging. In patients with early-stage disease, a radical hysterectomy can be performed with the fetus in situ. In locally advanced disease during the first trimester, abortion with evacuation of the conceptus is indicated. When surgical abortion is not feasible given the presence of a tumor obliterating the cervical OS, radiotherapy can be started with the intrauterine conceptus. This results in a miscarriage within three weeks. 1 3 Over 16 weeks, preference is given to feticide before starting treatment or evacuation.

### How should be the management of pregnant patients with stage IA1 or IA2 cervical cancer?

Conservative surgical treatment, such as conization, is recommended preferably between 14 and 22 weeks. After this gestational age, due to the risk of bleeding and pregnancy loss, quarterly surveillance should be carried out with colposcopy until delivery, and definitive treatment six weeks after delivery. Conization with high-frequency surgery is associated with less bleeding and complications. 1 8 The indication for cerclage is controversial.

### What is the role of lymphadenectomy in pregnant patients with cervical cancer?

Lymph node metastasis is one of the main prognostic factors in cervical cancer. For this reason, some authors advocate performing staging lymphadenectomy to truly determine the staging and prognosis and therefore, better select the candidates to continue the pregnancy. It is feasible up to 20 weeks, since after this gestational age, the uterine volume compromises the surgical field and the number of resected lymph nodes drops considerably, hence it is not considered appropriate for staging purposes. 1 3 The route of choice is laparoscopic in experienced hands, as it is associated with faster recovery and better postoperative pain control. Sentinel lymph node screening is not recommended in pregnant women given the risk of patent blue anaphylaxis and the lack of safety data with the use of technetium and indocyanine green during pregnancy. In case of positive lymph nodes, the tendency is to interrupt the pregnancy to allow standard treatment. Patients who refuse the interruption should be advised to undergo treatment with neoadjuvant chemotherapy performed until three weeks before delivery.

### How should be the management of pregnant patients up to 20 weeks and cervical cancer stages IB1 and IB2?

Several studies in patients with cervical cancer have shown a negligible risk of parametrial involvement when the pelvic lymph nodes are negative. 9 Therefore, by taking into account the significant morbidity of radical trachelectomy during pregnancy, such as pregnancy loss and bleeding, there is support in the literature to manage these patients with pelvic lymphadenectomy and wide conization or simple trachelectomy to obtain free margins, followed by cerclage. A multidisciplinary team, including radiologists, should perform the surgical planning with the aim of assessing the chance of resection of tumor free margin, maintaining a safe distance from the internal cervical os. In cases when a free-margin conization is not feasible, surgery is not recommended.

### How should be the management of pregnant patients over 20 weeks and diagnosis of cervical cancer stages IB1 and IB2?

The case series study of patients with a diagnosis of cervical cancer restricted to the cervix at the end of the second trimester and in the third trimester, who underwent expectant management with surveillance of progression showed excellent oncological outcomes, hence this treatment is an option. 8 In patients diagnosed at the beginning of pregnancy, or when expectant management is not considered prudent due to other prognostic factors (deep stromal invasion, angiolymphatic invasion or unfavorable histological types), neoadjuvant chemotherapy with carboplatin and paclitaxel is indicated every three weeks, starting after 14 weeks of pregnancy. If there is no progression, treatment should be carried out until 34/35 weeks to allow for full-term delivery. Given the risk of maternal and neonatal complications, such as infection and hemorrhage, chemotherapy should be discontinued three weeks before the planned date of delivery. 1 3

### How should be the management of pregnant patients with locally advanced tumor who wish to preserve the pregnancy?

Neoadjuvant chemotherapy with carboplatin and paclitaxel is indicated every three weeks, starting after 14 weeks of pregnancy. If there is no progression, treatment should be performed until 34/35 weeks and delivery at term. 10 Radiochemotherapy can be started two weeks after delivery. 1 3

### How should be the management of pregnant patients with stage IVB cervical cancer?

Palliative chemotherapy may be offered. Immunotherapies with recombinant humanized monoclonal antibodies such as bevacizumab and pembrolizumab are contraindicated during pregnancy. 1 2 3 Early referral to palliative care for control of pain and other symptoms is fundamental in the context of advanced and metastatic disease, contributing not only to improve the quality of life, but also to increase the survival of these patients.

### How should birth planning be in pregnant patients with cervical cancer?

In cases in which there is no progression of the disease or obstetric indication of anticipation of delivery, the ideal moment of delivery should be at the term of pregnancy. The mode of delivery is cesarean section when there is invasive cervical disease, with a corporal cesarean section to avoid the risk of extending the hysterotomy to the cervix and the consequent tumor laceration, with contamination of the abdominal cavity. 1 3 Vaginal delivery is contraindicated in patients with invasive cervical cancer, as it poses maternal and fetal risk. In addition to the risk of tumor bleeding and obstruction of the birth canal, the literature describes 20 cases of implantation in a laceration of the birth canal or episiotomy with a fatal outcome in most cases. 1 3 Arakawa et al. 11 reported two cases of children who developed squamous cell carcinoma of the lung after vaginal delivery in a patient with the same neoplasm in the uterine cervix. In patients treated with free-margin conization and without evidence of cervical disease, the mode of delivery is obstetric. 1 3

### How should definitive treatment be performed after childbirth?

The definitive treatment will depend on the patient's reproductive desire.

**Patients with reproductive desire:**

- Fertility-preserving treatment options can be offered to patients with stage up to IB1 and the surgical options are conization or radical trachelectomy with or without lymphadenectomy. The ideal time for surgery is four to six weeks after delivery.

**Patients without reproductive desire:**

- Patients with surgical treatment indication: Extrafascial or radical hysterectomy and pelvic lymphadenectomy can be performed right after the cesarean or six weeks later. The decision regarding the best moment for definitive surgical treatment must be individualized, taking into account the tumor biology, the patient's surgical risk and the surgeon's experience. Hysterectomy right after cesarean is associated with increased blood loss and perioperative complications, such as surgical wound infection and urinary tract infection. 12 In addition, sentinel lymph node biopsy is not feasible in this scenario. Waiting six weeks after delivery could allow this less morbid lymph node evaluation.
- Patients with indication of chemoradiotherapy can start treatment two weeks after delivery

## Final considerations

The concomitant diagnosis of cancer and pregnancy is a rare and dramatic situation. The medical literature is limited to case series and a consensus of the European Society of Gynecological Oncology/European Society for Medical Oncology (ESGO/ESMO); guidelines must always be interpreted with caution. Multidisciplinary and individualized evaluation is the best way to ensure the best outcome for the mother and, when there is a desire to preserve the pregnancy, for the fetus.

National Commission Specialized in Gynecologic Oncology of the Brazilian Federation of Gynecology and Obstetrics Associations (Febrasgo)

President:

Walquíria Quida Salles Pereira Primo

Vice-president:

Suzana Arenhart Pessini

Secretary:

Jesus Paula Carvalho

Members:

Angélica Nogueira Rodrigues

Caetano da Silva Cardial

Delzio Salgado Bicalho

Eduardo Batista Candido

Etelvino de Souza Trindade

Fernando Maluf

Francisco José Cândido dos Reis

Georgia Fontes Cintra

Marcia Luiza Appel Binda

Mirian Helena Hoeschl Abreu Macedo

Renato Moretti Marques

Ricardo dos Reis

Sophie Françoise Mauricette Derchain

Heloisa de Andrade Carvalho

Filomena Marino Carvalho

Aline Evangelista Albuquerque

Leandro Santos de Araújo Resende
